# Thyrotoxicosis

**Published:** 1941

**Authors:** A. Rendle Short

**Affiliations:** Surgeon, Bristol Royal Hospital; Professor of Surgery, University of Bristol


### The Bristol Medico-Chirurgical Journal

*"Scire est nescire, nisi id me
Scire alius sciret."*

SPRING, 1941.

### THYROTOXICOSIS.

BY

A. RENDLE SHORT, M.D., B.S., B.Sc., F.R.C.S.,

*Surgeon, Bristol Royal Hospital;*
*Professor of Surgery, University of Bristol.*

It would not be possible to deal at all fully with the scientific aspects of this subject in an article of reasonable length, and still less would it be possible to give a resumé of the enormous literature that has grown up concerning it. My purpose is much simpler; it is to present a record of personal experience and to offer suggestions as to the handling of these cases in general practice. The foundation for this study is a series of 488 patients operated on within the past seventeen years, and a considerable additional number seen but not operated on. Of these 371 were hospital cases, and 117 were treated privately.

The doctor's first concern is with diagnosis. He must be prepared to meet with a few cases in men, and a very few in children. Of my 488 patients, 424 were women, 64 were men, and two were girls under eighteen. One was only ten.

The ordinary case of thyrotoxicosis conforms so well to the familiar book picture that every doctor recognizes it at once. There are, of course, two types, with all gradations between them; the intermediate cases, in fact, are numerous. On the one hand, there are the patients with classical Graves's disease, usually women under thirty-five years of age, with exophthalmos, fatigue symptoms, quick pulse, loss of weight, quick fine tremor best seen in the extended fingers, and a general enlargement of the thyroid gland which came on at the same time as the toxic symptoms. Serious cardiac damage is unusual and the psychic symptoms are often well-marked. At the other extreme is the woman between forty and sixty, who has had a nodular goitre for many years,

B

which until recently has given little or no trouble. The tachycardia, tremor, fatigue symptoms and loss of flesh are as in the younger women, but there is no exophthalmos, and the psychic changes are usually less severe. The cardiac symptoms, on the other hand, are likely to be more serious, and auricular fibrillation is not uncommon. Some of the patients are bed-ridden invalids, with all the signs of chronic heart failure. It is customary to classify the first group as primary thyrotoxicosis, and the second as toxic adenoma or secondary thyrotoxicosis. As above remarked, many cases defy classification. Nevertheless the classification is worth preserving, for reasons of prognosis and also of treatment. Primary thyrotoxicosis may recover within a reasonable time, though quite frequently it does not; the question of operation is one for special consideration in each patient. Secondary thyrotoxicosis offers a threat of real danger to life, or very commonly of chronic invalidism; recovery under medical treatment is seldom seen unless a very long time can be spent in bed; and operation is nearly always indicated.

Difficulty in diagnosis may arise; thus, an obvious goitre may be unjustly blamed for symptoms of dysphagia due to cancer of the œsophagus, or for cyanosis and dyspnœa caused by myocardial degeneration. Much more commonly, no enlargement of the thyroid gland can be palpated, or a small but definite nodule is present, but is missed because it is not looked for with sufficient care, and yet the doctor may wonder, and justly, if the patient is suffering from thyrotoxicosis. Probably there is no such thing as thyroid poisoning without thyroid enlargement, but it need not be great, and it may lie buried beneath the muscles of the neck in such a way that nothing more of the gland than the normal can be felt through the skin. Or the source of the poisoning may be a nodule, a so-called "adenoma," lying in a slightly enlarged thyroid in such a position that it cannot be felt. Suspicion of thyrotoxicosis may be raised by the quick pulse and fatigue symptoms, or perhaps by psychic excitement either habitual or periodic. When auricular fibrillation supervenes in a person in middle life who has not had rheumatic fever or any other infection to account for it, thyroid poisoning is a very probable cause, and a hopeful one. I well remember the disgust of a man with a chronic rheumatic heart because the patient in the next bed, with very similar symptoms, was operated on for thyrotoxicosis and cured, while no such treatment was proposed for himself. Similarly, it may be possible to offer a quick and hopeful way to recovery for a difficult psychic patient by finding and removing a thyroid nodule, though considerable care is needed to pick the right case. The help of a psychiatrist may be valuable. The key to the diagnosis in cases of doubtful thyrotoxicosis is given by a careful palpation of the neck, by observing a significant combination of symptoms such as persistent tachycardia, made worse by excitement, along with tremor of the fingers and loss of flesh, and in the last resort by an estimation of the basal metabolic rate. Special apparatus and a skilled biochemist are necessary for this, and I have only resorted to it in cases of exceptional difficulty. Patients with excitement and tachycardia without thyroid enlargement and with a normal basal metabolism are probably suffering from an anxiety neurosis or other psychic disturbance. If the basal metabolic rate is increased, the thyroid is almost certainly the source of the trouble. It should be added that estimation of basal metabolism calls for meticulous care, without which some very surprising and anomalous results will be obtained.

Thyrotoxicosis may manifest itself in unusual ways. Persistent diarrhœa is one of these; glycosuria is another. Occasionally the symptoms are bizarre, such as seeing objects or people dancing before the eyes. Chronic headache is the main complaint with a few.

A few words must be said as to the natural prognosis of untreated or ill-treated cases. Many years ago Hale White published statistics of 161 hospital cases; 11 died in hospital, and of the survivors 54 per cent. were cured, 25 per cent. better, 5 per cent. no better, and 16 per cent. died not long afterwards. Figures for private patients were similar. Several other published end-results are to much the same effect. Cases that come on rapidly often recover quickly. Usually the best clue to the course the disease is taking is the body weight; a decrease means getting worse, and an increase means getting better. These tables are old, and probably include a large proportion of the younger women with primary thyrotoxicosis or classical Graves's disease. If the older women, so many of whom are seen in hospital to-day with secondary thyrotoxicosis, had been included, the number of patients classed as "no better" would have been much larger. In Hale White's time, the thyroid origin of many cases of chronic cardiac failure was not recognised, unless the gland happened to be quite large. Patients with secondary thyrotoxicosis are not likely to get well. They drift into chronic invalidism.

To what extent can the outlook be improved by medical treatment, or by radiotherapy? Prolonged rest in bed, of course, is most valuable. Lugol's iodine, twenty or thirty minims a day, will usually produce considerable improvement, especially in cases of primary thyrotoxicosis, but it is often wrongly given. Its usefulness is exhausted after two or three weeks, and it ought to be stopped. Lugol's iodine is very useful to the surgeon in the pre-operative period, but unfortunately in only too many cases the drug has been given for a long time and has lost its virtue. It may be necessary to postpone operation for three months so that its help may once more be obtained to make the operation safer, and it is difficult to explain to the patient the reason why, without seeming to blame the doctor. When Lugol's iodine has been stopped, bromides and belladonna may be given, or a number of other remedies that are in favour with one physician or another. In cases of tachycardia with auricular fibrillation digitalis is of course very valuable to slow down and rest the heart. With regard to radiotherapy, whether with X-rays or radium, I remember asking Dr. Berven, at the Stockholm Radium-hemmet, his opinion ; and he told me that he advised surgery in the ordinary case, and radiotherapy for mild degrees of the disease, and for those who are very gravely ill. This may be taken as sound practice. We have not much experience of radiotherapy here, but as far as it goes it confirms that improvement may be expected, after a course lasting several months, but not a cure.

There are two principal drawbacks in the way of medical treatment. One is the length of time that must be spent in bed, or at any rate away from active employment. For primary thyrotoxicosis this will probably mean three months in bed and another three months off work. At the end of this tedious course, there is only a fifty-five per cent. probability that the patient will be cured, and some of the alleged cures relapse when life becomes strenuous again. It will be observed that no inconsiderable number of patients die. For secondary thyrotoxicosis, the period of rest must be longer still, and the prospects of cure decidedly less.

In patients with much exophthalmos, there is another grievous danger, which I have seen once or twice in patients medically treated. Corneal ulceration may occur, followed by hypopyon and blindness in one or both eyes. Nevertheless, I believe that trial should be made of medical treatment in all cases, even if it proves to be only a preliminary to surgery. In certain favourable cases a serious attempt should be made to effect a medical cure. I refer to the young woman, or child, with primary thyrotoxicosis, not of severe degree and of less than six months' duration, whose home circumstances are such that time can be spared without economic disaster. Patients under forty who have mild hyperthyroidism, but are well able to do their work without distress, need not be advised to resort to surgery. And lastly there are a very few neglected cases, with big goitres and bad hearts, or with very vascular pulsating thyroids, who cannot be made safe for operation, and are probably better let alone, or advised a course of radiotherapy and rest. I have only once refused operation for a patient of this type, but it would have been wiser to have refused a few more, in my earlier years of thyroid surgery. Two or three have been advised to come in for thyroidectomy, but have died whilst being prepared in the wards.

What are the risks and benefits of thyroidectomy ? Ought the gland to be removed in one stage, or two, or more ?

In the four years 1937 to 1940, including January, 1941, I operated on exactly 200 cases, three of whom died in hospital. None of my last 66 cases operated on in private has ended fatally. Two patients at the Bristol Royal Infirmary who had a thyroidectomy performed for severe cardiac failure not due to thyrotoxicosis are not included ; both died within a few days.

Prior to 1936 the mortality was decidedly higher, for instance in the period 1925–1930, it was 7·6 per cent. This was partly because a relatively large number of " bad starters " were included. Some had enormous vascular goitres ; in other cases the heart was worn out. Two patients were found at autopsy to be afflicted with unsuspected pyonephrosis. As above remarked, a good many of these cases could never have been made safe for surgery. Nowadays not many such neglected cases are left in the district ; they are nearly all well or dead.

I hope and believe, however, that the fall in the operative mortality is largely due to improved surgical technique and better methods of anæsthesia. This paper is not written specially for surgeons, and it is not proposed to go into detail, but a few points may be mentioned. The anæsthesia in nearly all my cases has been in the hands of Dr. Leonard Moore, to whom I am greatly indebted. After trying a number of methods, several of them over a period of years, we have come to regard local anæsthesia with half per cent. novocaine and a few drops of adrenaline solution, combined with basal narcosis in the form of morphine and scopolamine, as safest and best. The patient is given morphine gr. $\frac{1}{4}$ and scopolamine gr. 1/100 an hour before the time fixed for the operation, and if she comes to the table quite lively, another sixth of a grain of morphine is administered. We are able to guarantee that the patient will not feel any pain, and in fact many of them sleep through the later part of the operation, though they can usually be roused to cough and strain, to test hæmostasis, just before the wound is closed. A few very frightened people are given gas and oxygen as well, especially if the thyrotoxicosis is of mild degree.

Thyroidectomy is an operation beset by many dangers, of which the earliest, chief, and most controllable is hæmorrhage, usually coming on about four hours after the patient has returned to bed. The ordinary surgical precautions against reactionary bleeding are quite inadequate in thyroid cases. I now tie all vessels with fine silk, not catgut. Catgut knots are insecure, and one may see them fly off the vessels in the most alarming manner when the patient strains. Also, catgut irritates the tissues and silk does not. All important vessels ought to be stitched, not merely tied. At the end of the removal stage the patient must be made to cough and strain. Drainage is provided for twenty-four hours. It is most important to have a special nurse to watch the patient closely till the danger period is passed. One reason why we gave up avertin, and later paraldehyde per rectum, as basal narcotics, is because the patients are apt to be restless during the danger period, whereas morphine keeps them quiet for hours.

A second danger is damage to the recurrent laryngeal nerve, especially on the right side. It is to be avoided by leaving a strip of thyroid tissue posteriorly and close to the trachea, but even then the nerve may be stretched by pulling, though it is not divided. Husky voice, inability to sing, goose-cough, and a certain amount of shortness of breath on exertion are the symptoms of injury to the nerve, and it is very difficult to remedy it. Fortunately, most of the cases improve in time, even as late as six or nine months after operation. Bilateral damage is very serious and may prove fatal from laryngeal obstruction. There is one unhappy example of this amongst my earlier cases. A third danger is removal of too much parathyroid tissue, resulting in tetany. This complication comes on quite unexpectedly in spite of the fact that no deviation from the normal technique has been made. Apparently in some persons the parathyroid glands lie embedded in the thyroid and removal with the thyroid is more or less inevitable. Half a dozen of my patients have had a transient tetany in the first few days after operation ; the first sign of it is a complaint of tingling all over, then the hands and feet go into spasm. In two patients the liability to tetanic spasms persisted for a year or more, but eventually they got well. The treatment is to give plenty of calcium, extra milk, and dilute hydrochloric acid. During the spasms calcium gluconate by injection is indicated ; it usually gives prompt relief. In difficult cases, A.T.10 (dihydrotachysterol) given daily by mouth is very efficient, but it is expensive and difficult to get. The same may be said of parathormone.

A certain number of patients die during the first few days of pulmonary complications, or of cardiac failure. In the beginnings of surgery for thyrotoxicosis, the great danger was acute hyperthyroidism coming on within twelve or twenty-four hours of the operation. It is probably due to squeezing the gland, and to absorption from open vessels on cut surfaces, though this has been challenged. The patient may be wildly excited, may suffer from a tremor that shakes the bed, the pulse becomes uncountable, and the temperature is above 104°. These severe reactions are seldom if ever seen in patients properly prepared, though the majority do show a quickening of the pulse, and a temperature around 100°, on the second, third and fourth days.

This brings us to the vitally important question of proper preparation before operation. All cases need five to seven days in bed in the hospital or nursing home before the ordeal. During this time proper sleep must be secured, the patient watched for catarrhal symptoms, and Lugol's iodine given. It is a great pity if this valuable remedy has been thrown out of action by using it too long at home. Patients with severe thyrotoxicosis or with damaged heart muscle need a much longer period of preparation, preferably under the care of a physician specially interested in such cases. Some of them will relieve the surgeon's mortality-rate by dying before they come to him. Post-operative thyrotoxicosis is treated by administering the greatest possible quantity of fluids by all routes, by continuing Lugol's iodine, by keeping the patient quiet with morphine or diamorphine, and in the worst cases b intravenous sodium iodide. If the temperature is above 104°, th patient should be sponged.

Much has been written or said on the question of one-stage or multiple-stage thyroidectomy. It is theoretically possible to remove the gland by six stages, namely, ligature of the right superior thyroid artery, then the left, then the right and left inferior thyroid arteries, then removal of the right lobe, and finally of the left. Personally, I have not gone beyond three stages, but up till 1936 many of my severer cases were two-stage operations. Since 1936 nearly all my thyroidectomies have been done as a single complete operation. This does not always mean removal of both lobes ; for a few toxic goitres all that is necessary is to enucleate the large nodule that has undergone some change and is pouring the toxin into the blood. I think that post-operative hyperthyroidism is less dangerous when there is very little thyroid gland left to furnish the poison. Normally about seven-eights of both lobes is to be removed, and the whole of the isthmus. A hemi-thyroidectomy may still occasionally be the safest when there is a good deal of blood lost ; the vessels supplying some thyroids are enormous, and if one gets torn or lost the bleeding may be furious. For some years I made it a routine to check the blood lost by weighing the swabs, but by experience I came to be able to make a fairly exact estimate. One ought to stop if the loss reaches a pint. In most cases, of course, the hæmorrhage is trivial, every artery and vein being seen and secured before it is cut.

The secret of safety in treating thyrotoxicosis is to remove the gland before the heart muscle has suffered much organic damage, as it assuredly will, in middle-aged people, if it is driven too fast year after year. If these neglected cases are excluded, the death-rate before and after operation should not exceed 0·5 per cent., if the necessary care is taken over hæmostasis. Unfortunately, there is a type of woman who from timidity, or from unselfish devotion to her family, will not consult her doctor until she is really prostrate ; and there is a type of doctor who advises medical rather than surgical treatment because he remembers that twenty-five years ago thyroidectomy for Graves's disease was reckoned dangerous. Consequently we still see a number of patients with auricular fibrillation due to thyroid poisoning, and their only hope of returning to normal life and activity is a successful thyroidectomy. Auricular fibrillation has been noted in 19 of my cases since 1929, but no doubt it was as a matter of fact present temporarily, if not constantly, in a good many others. Of these 19 patients, 15 came through the operation period successfully, and nearly all of them went home no longer fibrillating. To secure this happy result, however, surgery may not be sufficient. It has been my custom to ask Dr. Herapath, who has helped me greatly over many years with difficult cases both before and after operation, to come in and prescribe quinidine. The heart "jumps straight" after the first or second dose as a rule, but one woman needed five doses. Quinidine is reputed to be dangerous, but we have not yet seen it do any harm.

Lastly, we have to consider the end-results of thyroidectomy. Information concerning 113 patients has been obtained, from one to ten years after operation. Most of them have been seen and examined; some were written to; the rest have been reported on by the family doctor. The cases are in no way selected; some had severe symptoms when they first came up; in a few the thyrotoxicosis was slight though there was an obvious goitre. Inasmuch as we do not usually hear again from satisfied patients, whereas those with a poor result are likely to return to ask for further treatment, it is perhaps fair to conclude that the follow-up tends to exaggerate the number of failures and to hide the successes. In war-time, there are many who cannot be traced. The results were as follows: Seventy-six (67 per cent.) had an excellent result. Doctors are often enthusiastic about it. The patient puts on weight, almost too much at first, but it drops to normal when they get active; their tempers improve, and they become most useful members of the family, with a quiet pulse and no tremor. Exophthalmos improves, but not as much as the other symptoms. Thirty (26·5 per cent.) were better, often much better, but still had some complaints, sometimes quite unconnected with the disease or operation. Three (2·6 per cent.) patients were no better, and four (3·5 per cent.) died months or years after going home. One of these had a stroke, another died of cardiac failure. In two cases the cause of death is unknown.

The following is an example of a case which is classed as "Better, but not cured" after operation. Mrs. R., aged 48, was seen in April, 1937, with Dr. Healy. She had had typical symptoms of thyrotoxicosis for four years. In 1936 she was seen by a consulting physician for myocardial degeneration, ascites and œdema of the feet, and was told she was too ill for operation. She was a bed-ridden invalid for over a year. In 1937 she had auricular fibrillation, with huge ascites and some œdema of the feet. The thyroid was moderately enlarged. The ascites was tapped twice; then in June the right half of the thyroid gland was removed. In October she was tapped again, and a week later the left half of the thyroid was removed. There was a smooth convalescence.

Her doctor saw her in March, 1941. He writes as follows: "I have seen her to-day and found her condition to be very good. Approximate gain in weight 2½ stone (9 st. to 11 st. 6 lbs.). Pulse after exertion, 80. Still some evidence of enlarged heart, but compensation established. Capable of doing *all* her own housework, including the heavier tasks. Sleeps well (even during air-raids), eats well, and is a much happier woman. She has recently prepared her own garden for spring planting. She still suffers from a peculiar form of agoraphobia, which brings on diarrhœa on any attempt to appear in public, *e.g.*, she dare not attempt to walk down the main road without disastrous consequences, but can walk four or five miles in country without distress."

The patients who still had some grounds for complaint fall into various groups. It is often a problem to know just how much thyroid tissue to remove, and how much to leave. If the symptoms appear to be caused by one or more nodules, so-called adenomata, these are removed and the rest of the gland left. In a few cases, too much has been taken. A transient myxœdema, lasting a few weeks, is not uncommon. Only in two cases has there been a persistent myxœdema sufficiently pronounced to call for the administration of thyroid. Much more frequently, and especially when the thyroid enlargement has been inconsiderable, not enough has been taken, and some thyrotoxicosis has persisted, or has returned a few years later. In 43 of my patients the thyroidectomy was deliberately two-staged, but in addition to these there were 7 cases who had to have a further removal, and in 3 of these a third operation was necessary, spread out over a period of ten years or thereabouts. In some of these the first operation was performed by another surgeon.

Recurrent laryngeal paralysis, leading to hoarseness, inability to sing, and dyspnœa on exertion, is troublesome, and difficult to avoid. The notes of my hospital cases are not always reliable in this respect, but of my 117 private patients 3 went home with some signs of paralysis of a vocal cord. As already mentioned, most patients with symptoms of paralysed cord recover in the course of a few months, but not all.

Although several patients had tetany in the first few days after operation, it only persisted long enough to be serious in two patients, both hospital cases, and in these it eventually cleared up. There was one curious example of recurrence. A patient who had never had tetany before developed a sharp attack several years after her thyroidectomy, when she was starved for three days in preparation for a gynæcological operation. No doubt she ran short of calcium.

Patients with hyperthyroidism are fundamentally nervous subjects as a rule, and the operation does not induce in them the temperament of an old tabby cat. It is not surprising therefore that a good many of them still complain of one thing or another, unconnected with the thyroid, or with the operation, so that they cannot be classed as perfect cures.

A few words may be added about the results of thyroidectomy in special cases. In the private series glycosuria had been noted in four cases ; it cleared up after operation. One had a goose-cough from pressure on the laryngeal nerve ; this also cleared up. Another had asthma as well as thyrotoxicosis ; the attacks were made fewer and milder. In half a dozen cases the symptoms were mostly psychic ; these usually improved. One patient had periodic attacks of severe psychotic upset in which she was virtually insane ; these were reduced to about one a year.

Two patients were children, aged 10 and 13. Both were very severe cases ; one had been reduced to a skeleton in spite of much medical treatment in hospital. Thyroidectomy had to be carried out in her case in three stages. She did very well, and has continued in good health for seven years. The other child died three weeks after operation.

Finally, how may the results of thyroidectomy be improved in the future ? A custom has grown up in most parts of the country, and indeed all over the world, of sending these cases to a certain small number of surgeons who devote particular attention to the many problems they present, and who are able therefore constantly to improve their technique. This makes for progress in thyroid surgery, as in every other branch of the healing art. It is even more important for obtaining better results that doctors should make an early diagnosis, even when the thyroid enlargement takes careful looking for, and that not too many months should be allowed to slip by before it is discovered which patients are going to be cured by medical treatment, and which not. In the near future I trust we shall no longer see patients whose hearts have been exhausted by years of fast driving. Take these, and the enormous vascular goitres, and a few hyper-acute cases, out of our operating lists, and the surgery of thyrotoxicosis becomes almost absolutely safe, and seldom fails to give very great relief.

### SUMMARY.

1. A report is presented of 371 hospital and 117 private cases, 488 in all.

2. Thyrotoxicosis is not infrequently the cause of a patient's fatigue symptoms, or tachycardia, or "nervousness," although the palpable thyroid enlargement is very slight. The gland lies buried beneath the muscles.

3. Certain early cases of primary thyrotoxicosis, classical Graves's disease, are suitable for medical treatment.

4. The tendency to administer Lugol's iodine for months on end is unfortunate. Courses should be brief.

5. The operation mortality in this series used to be 7·6 per cent. In the last 200 cases, it has been reduced to 1·5 per cent. Of 19 patients with auricular fibrillation, 4 died.

6. Of 113 followed up, 67·2 per cent. had an excellent result ; 26·5 per cent. were better, often much better ; 2·6 per cent. had a poor result ; and 3·5 per cent. died at home. The incidence of tetany, and of vocal cord paralysis, is discussed.

7. To secure better results, patients must be diagnosed and sent for treatment before the heart is worn out by years of fast driving.

